# Dengue Human Infection Models Supporting Drug Development

**DOI:** 10.1093/infdis/jiu062

**Published:** 2014-06-15

**Authors:** James Whitehorn, Vinh Chau Nguyen Van, Cameron P. Simmons

**Affiliations:** 1 Department of Clinical Research, London School of Hygiene and Tropical Medicine; 2 Centre for Tropical Medicine, Oxford University, United Kingdom; 3 Oxford University Clinical Research Unit, Hospital for Tropical Diseases; 4 Directorate, Hospital for Tropical Diseases, Ho Chi Minh City, Vietnam; 5 Nossal Institute for Global Health and Department of Microbiology and Immunology, University of Melbourne, Parkville, Victoria, Australia

**Keywords:** dengue, human infection model, clinical trial, drug development

## Abstract

Dengue is a arboviral infection that represents a major global health burden. There is an unmet need for effective dengue therapeutics to reduce symptoms, duration of illness and incidence of severe complications. Here, we consider the merits of a dengue human infection model (DHIM) for drug development. A DHIM could allow experimentally controlled studies of candidate therapeutics in preselected susceptible volunteers, potentially using smaller sample sizes than trials that recruited patients with dengue in an endemic country. In addition, the DHIM would assist the conduct of intensive pharmacokinetic and basic research investigations and aid in determining optimal drug dosage. Furthermore, a DHIM could help establish proof of concept that chemoprophylaxis against dengue is feasible. The key challenge in developing the DHIM for drug development is to ensure the model reliably replicates the typical clinical and laboratory features of naturally acquired, symptomatic dengue.

The 4 serotypes of dengue virus (DENV-1 to DENV-4) are the most important arboviral pathogens of humans. The global burden of dengue, recently estimated at approximately 100 million cases per year, remains unchallenged by licensed vaccines or sustainable vector control strategies [1]. The scale of the dengue burden in the endemic countries of Asia and Latin America has a negative economic impact and places a significant clinical burden on often fragile health care systems [2–4].

Dengue is an acute systemic febrile illness that manifests with abrupt onset as an undifferentiated fever that is difficult to distinguish from other infections without laboratory diagnostic tests [1]. Headache, malaise, myalgia, retro-orbital pain are common symptoms in adults [5]. The clinical features differ by age group, with symptoms such as cough, vomiting and abdominal pain more common in children [6]. Leukopenia and thrombocytopenia are common laboratory features.

In most cases, fever and symptoms resolve uneventfully after 3–7 days with no long-term sequelae [1]. In a subset of cases, a transient vascular leakage syndrome develops after 3–4 days of illness, which, when severe, can lead to life-threatening hypovolemic shock (called dengue shock syndrome) and/or hemorrhage [1, 5]. The vascular leak syndrome may be associated with complement activation and a coagulopathy that can contribute to the risk of hemorrhage [7, 8]. Signs of vascular leakage include an increasing hematocrit, microalbuminuria, or the accumulation of fluids at serosal surfaces (eg, pleural effusions) [5].

Supportive care and the careful titration of minimum volumes of parenteral crystalloid fluids to maintain stable cardiac output during the 1–3-day period of vascular permeability are critical elements in dengue case management [5]. Vasopressors are commonly used in cases with prolonged shock. Clinically significant hemorrhage requiring use of blood products is more common in adults than in children [9].

Improvements in the management of severe dengue cases have seen the case mortality rate decline over the last decade in many but not all endemic settings [10–12]. At present there is no validated way of identifying which patients will progress to more severe disease, meaning that health facilities in endemic areas are often overwhelmed with patients with dengue who require ongoing observation as outpatients or are admitted for inpatient observation.

## THE CASES FOR DENGUE THERAPEUTIC AND PROPHYLACTIC AGENTS

### Antivirals

A drug that can shorten the duration of illness and reduce the risk of disease progression would be a significant advance for both patients and for overstretched health systems in dengue-endemic countries. An obvious approach is to target virus replication with a small molecule antiviral drug that given early in the course of illness has the potential to shorten the duration of infection. Moreover, because high early DENV viremia levels are a risk factor for the development of more severe disease, an antiviral drug might also reduce the incidence of severe complications [13, 14]. In addition, a drug that reduces the magnitude and duration of viremia has the potential to reduce onward DENV transmission by reducing the length of time an individual patient is infectious to mosquitoes [15]. Alternatives to small molecule antiviral drugs include therapeutic monoclonal antibodies; however, high costs and requirement for parenteral administration are currently barriers to their use.

### Disease Modulators

Both host and virus factors are important in influencing the outcome of DENV infection. A range of host factors, such as age, sex, genotype, and flavivirus infection history, influence the disease outcome [10, 16, 17]. Because the manifestations of severe dengue are typically observed in secondary DENV infections and when innate and adaptive immune responses have driven steep declines in viremia levels, it is widely held that the immune system plays an important role in disease pathogenesis [18]. This represents the rationale for use of an immunomodulatory drug that can favorably modulate the poorly defined immunopathogenic mechanisms postulated to underlie severe dengue. It is possible that the dichotomous approach of targeting either the virus or the immune response is an oversimplified strategy to the development of dengue therapeutics. Other physiological systems are involved in the control of endothelial integrity, but their role in dengue remains poorly understood [19].

### Prophylaxis

A vaccine would be the most effective prophylactic public health intervention for control of dengue, but the development of an efficacious tetravalent dengue vaccine is proving to be a challenge [20, 21]. An alternative or adjunctive approach is the use of drug chemoprophylaxis in the community. Chemoprophylaxis using oseltamivir has been advocated for controlling the spread of influenza within households and was commonly used in some settings during the 2009 H1N1 influenza pandemic, creating a precedent for such an approach for an acute viral infection [22, 23]. The safety profile of a prophylactic dengue drug would have to be exemplary, and there are major questions of whether this approach could be cost-effective and sustainable, given the temporally and spatially heterogenous patterns of DENV transmission in endemic regions [24, 25]. Moreover, in most dengue-endemic regions a significant fraction of the population is already immune and will not benefit from chemoprophylaxis. Consequently, in endemic areas the number needed to treat, and the duration they need to be treated for, may be large for the prevention of a single symptomatic case and very large for the prevention of severe cases. There is a marginally stronger argument for chemoprophylaxis in populations with no immunity to DENV. Examples of this include dengue-naive travelers or military personnel who are transiently present in areas of high DENV transmission. Similarly, chemoprophylaxis could be indicated for communities experiencing “virgin soil” dengue outbreaks because of importation by viremic travelers or infected mosquitoes.

## PREVIOUS CLINICAL TRIALS OF THERAPEUTICS FOR DENGUE AND RELEVANCE TO THE DENGUE HUMAN INFECTION MODEL

For a disease with an estimated global burden prevalence of 100 million cases per year, remarkably few clinical trials of treatment approaches have been performed. Early trials tested fluid resuscitation approaches in patients with hypovolemic shock [26–28]. More-recent trials have tested the candidate antiviral agents chloroquine and balapiravir or have targeted the immune response by use of oral corticosteroids [29–31]. Although none of these trials have demonstrated therapeutic efficacy, they have provided a framework for the conduct of such trials and has led to the publication of recommendations for a standardized approach to dengue clinical trial design and conduct [15]. In addition, a trial of lovastatin in dengue is currently underway and further trials are planned [15, 32]. The virological findings in these trials, consistent with previous literature, have underscored the need for antiviral therapy to commence early in the course of illness, because illness resolves and patients are no longer infectious to *Aedes aegypti* mosquitoes by 5–7 days after illness onset [14, 15, 33–35].

## POTENTIAL ROLES FOR A HUMAN INFECTION MODEL IN DENGUE DRUG DEVELOPMENT

In principal, a dengue human infection model (DHIM) provides opportunities for fast-tracking dengue drug development, particularly therapeutic and prophylactic antivirals. The overall strength of the DHIM is that it allows for controlled prophylactic or therapeutic studies of DENV infection in susceptible participants who have been preselected for a set of desirable characteristics. In contrast, prophylactic or therapeutic studies of DENV infection in an endemic country population are subject to the vagaries of ethnic diversity of the patient population, fluctuating serotype and genotype prevalence, variable viremia levels, and prior flavivirus infection histories. Collectively, these sources of variation in an endemic country population probably boost the required patient sample size required for detection of therapeutic or clinical safety signals.

## CRITICAL HURDLES FOR A DHIM

Central to the usefulness of a DHIM is whether it mimics the main features of a symptomatic dengue case. This is a fundamental and critical hurdle in the development of a successful DHIM for use in dengue therapeutic development. Thus, the kinetics of clinical, virological, hematological, and biochemical changes that are typically present in a naturally acquired and clinically uncomplicated adult dengue case should also manifest in the adult DHIM. In the beginning, at least, we assume that the DHIM will involve participants who are flavivirus naive, and thus all experimental infections will be primary infections. In the context of dengue drug development, the characteristics of a symptomatic, naturally acquired DENV infection that a DHIM would need to replicate include acquisition of infection and certain clinical, virological, and laboratory features.

### Acquisition of Infection

A DHIM could use DENV-infected mosquitoes to elicit infection in human volunteers. Although this approach may be optimal in replicating the natural history of DENV infection because, for example, the immunoregulatory effects of mosquito saliva would be replicated, it may present additional regulatory and logistical hurdles. Alternatively, a DHIM could use subcutaneous inoculation of laboratory-cultured virus, as used in the challenge experiments by Sun and colleagues [36]. This approach bypasses issues surrounding use of mosquitoes, although it remains to be determined if there are material differences between needle-delivered and mosquito-delivered DENV infection.

### Clinical Features

Typical signs and symptoms of dengue that a DHIM would need to replicate in otherwise healthy adults include fever, headache, myalgia, musculoskeletal pain, and nausea or vomiting [37]. Minor hemorrhagic features may occur between days 3 and 6 of illness (eg, petechiae) [37]. Prevention or rapid amelioration of these self-limiting symptoms will be a target of dengue therapeutic drug development. Primary infections in otherwise healthy adults are rarely associated with severe clinical complications, but it would be prudent to exclude the elderly and those with comorbid conditions from the volunteer pool, because these groups are at elevated risk of severe complications [38, 39].

### Virological Features

The intrinsic incubation period of DENV infection has been estimated to be between 4–10 days [5]. Work that reanalyzed historical mosquito exposure experiments estimated that the intrinsic incubation period in primary infection was approximately 6 days [40]. The duration of viremia varies by serotype and DENV infection history. The peak viremia level is often not observed in naturally occurring infections because it precedes the time point when patients attend health care facilities, but findings suggest that the peak level occurs later in primary infections as compared to secondary [33]. Studies suggest that primary infections with DENV-1 are associated with a longer viremic period and higher viremia levels [33, 41]. An optimal DHIM would replicate the virological features of a naturally acquired DENV infection to enable confident predictions of therapeutic or prophylactic drug efficacy in the field.

### Laboratory Features

A DHIM should elicit thrombocytopenia and leukopenia, both typical features in patients with primary or secondary dengue. In addition, many patients have minor elevations in transaminase and creatine kinase levels [9, 42]. Clotting abnormalities are also often observed in severe cases with elevated activated partial thromboplastin times and reduced levels of fibrinogen; however, these occur less frequently in primary infections [43].

## STRENGTHS AND WEAKNESSES OF A DHIM FOR DRUG DEVELOPMENT

The strengths and weaknesses of a DHIM for performing early-phase clinical studies compared to performing such studies in naturally acquired cases in an endemic country are summarized in Table 1. In short, the great advantage of the DHIM for early-phase drug development is the ability to perform controlled prophylactic or early therapeutic studies in a setting amenable to intensive pharmacological and basic research investigations.

**Table 1.** Early-Phase Clinical Studies of Antiviral Drugs: Strengths and Weaknesses of the DHIM vs Trial Conducted in a Dengue-Endemic Setting

Hurdle	DHIM	Dengue-Endemic Setting	Notes
Dengue case burden	At call	Usually seasonal and with yearly variation in case numbers	In endemic settings dependent on the force of infection
Clinical research capacity	Available	Variable	In endemic settings dependent on local expertise, experience and resources
Efficiency of regulatory system	Variable	Variable	In endemic settings dependent on research infrastructure and local regulatory requirements
Accredited testing laboratories	Available	Variable	In endemic settings dependent on research infrastructure and local regulatory requirements
Ability to ship research specimens across international borders	Yes	Variable	In endemic settings dependent on research infrastructure and local regulatory requirements
Variance in viremia and serotypes	Serotype controlled; possibly less variance in viremia	Variable	In endemic settings dependent on local viral epidemiology
Early therapy possible	Yes	Variable	In endemic settings dependent on patients presenting early
Pharmacokinetic studies	Yes	Yes/Variable	May require international shipping of samples from endemic countries
Intensive pathophysiological monitoring	Yes	Possibly	Ability to conduct intensive monitoring is dependent on the local resources available
Chemoprophylaxis studies	Yes	More difficult	The controlled setting of the DHIM is ideally suited to this kind of study
Provide proof of concept that therapy reduces major clinical complications	Probably not, requires large sample sizes	Yes	Severe clinical events in healthy adults with primary DENV infections are rare
Provide proof of concept that therapy mitigates typical lab features	Probably not, requires large sample sizes	Yes	Demonstrating mitigation would require large sample size
Serious adverse events; clinical experience of managing severe dengue	Variable	Yes	Extensive clinical experience and expertise in endemic settings
Clinical studies of secondary heterotypic DENV infections	Difficult	Yes	Ethical hurdles for the DHIM in view of known risks associated with secondary heterotypic infections
Clinical studies in participants who have received dengue vaccine candidates	Yes	Yes	In the DHIM will depend on the balance of perceived risks and benefits

Abbreviations: DENV, dengue virus; DHIM, dengue human infection model.

## FOCUS AREAS IN WHICH THE DHIM COULD SUBSTANTIALLY ADVANCE CLINICAL DRUG DEVELOPMENT

A DHIM that mimics natural infection would be particularly well suited to early-phase studies of chemoprophylaxis and accompanying pharmacokinetic studies, in which the timing of treatment and infection can be controlled. In contrast, in a dengue-endemic country, early-phase chemoprophylaxis trials would require large and lengthy community-based cohort studies, as has been used to derive evidence for oseltamivir in the chemoprophylaxis of influenza [22]. The DHIM would thus be a more cost-effective and time-efficient route to acquiring proof of concept evidence that chemoprophylaxis for dengue is possible. For a given antiviral drug, a DHIM would also be well-suited for studies that accurately define the therapeutic window in symptomatic cases, since the time of infection, onset of symptoms and treatment will be prospectively documented. In contrast, in endemic countries enrollment of study participants is dependent on patients seeking health care, who will have various durations of illness history at the time of study enrollment. A DHIM can also aid the development of alternative therapeutic approaches, such as immunomodulatory agents. In addition, a DHIM may aid the development of noninvasive physiological monitoring techniques within clinical trials, perhaps through the identification of appropriate physiological measurements that could be used as end points [15].

In conclusion, the work of Sun and colleagues [36] has shown that a DHIM is possible. This is a significant advance in the field with the potential to contribute to the development of successful dengue therapeutics and much else. The future use of the DHIM in dengue clinical research will require a significant investment from multiple stakeholders. Its contribution in drug development may aid in the selection of appropriate candidate drugs for further development and assist pharmacokinetic studies. The potential significance of these contributions suggests that investing in a DHIM is both appropriate and timely.
